# Editorial: Advances in Pediatric Hematopoietic Cell Therapies and Transplantation

**DOI:** 10.3389/fped.2022.847288

**Published:** 2022-01-28

**Authors:** Emmanuel Katsanis, Patrick J. Hanley, Richard J. Simpson

**Affiliations:** ^1^Department of Pediatrics, University of Arizona, Tucson, AZ, United States; ^2^Center for Cancer and Immunology Research, Children's National Hospital, The George Washington University, Washington, DC, United States; ^3^School of Nutritional Sciences and Wellness, University of Arizona, Tucson, AZ, United States

**Keywords:** transplant, hematopoietic, cell therapeutic, leukemia, haploidentical hematopoietic stem cell transplantation

Hematopoietic cell transplantation (HCT) offers curative treatment for numerous malignant and non-malignant disorders and has evolved into a relatively safe procedure with reduced transplant related mortality. There have been notable advances in HCT in recent years. Undoubtedly one of the most significant has been the re-emergence of haploidentical (haplo) HCT which has rapidly evolved into a comparable alternative to unrelated donor transplantation (1, 2). However, haplo-HCT has not been as widely adopted in pediatrics in part due to the option of umbilical cord blood (UCB) transplantation that younger patients may have (3). T-cell replete haplo-HCT with post-transplant cyclophosphamide (PT-CY) is the most frequently applied platform, while αβ T-cell and CD19^+^ B-cell depletion is utilized in some pediatric European centers (4). In the accompanied collection of publications, Sano et al. report their experience in 19 pediatric patients with relapsed/refractory acute lymphoblastic leukemia receiving T-cell replete peripheral blood stem cell (PBSC) haplo-transplantation following total body irradiation (TBI) or busulfan (BU) based myeloablative conditioning (MAC) in 89% of patients. Thymoglobulin, tacrolimus, methotrexate and prednisolone were used as graft-versus-host disease (GvHD) prophylaxis. Not unexpectedly and partially due to the high CD3 cell dose infused, the incidence of grade II-IV and III-IV acute and chronic GvHD was unacceptably high, 100, 50, and 67%, respectively. Nonetheless, they demonstrated an encouraging 3-year overall survival of 75% in patients in complete remission (CR) and 45% in those not in remission at the time of transplantation, whilst acknowledging that their results should be interpreted with caution due to their small numbers. Katsanis et al. updated their pediatric haplo-BMT experience of 21 patients with hematologic malignancies (89% in CR) using TBI or BU based MAC followed by T-replete haplo-BMT with PT-CY or PT-CY/bendamustine (5). Grade II-IV and III-IV acute and chronic GvHD were manageable at 30, 15, and 18%, respectively. With a median follow-up >2 years, their outcomes were excellent with OS of 84%, progression free survival (PFS) 74% and graft-versus-host disease relapse-free survival (GRFS) of 50%. Gupta and Wagner review the current status of UCB transplantation for malignant and non-malignant disorders. They also discuss strategies to enhance hematopoietic recovery as well as alternative uses of UCB such as for the generation of virus-specific T cells, T regulatory cells for prevention of immune reactivity, and NK cell therapy for hematologic malignancies.

Supportive care pre-, during and post-HCT is of critical importance in reducing adverse drug effects, organ toxicity, infectious complications and ultimately non-relapse mortality. The next series of articles address some of these important issues. A report by Ifversen et al. from the Supportive Care of the Pediatric Diseases Working Party (PDWP) of the European Society for Blood and Marrow Transplantation (EBMT) provide an updated and comprehensive set of recommendations of infection prevention for children and young adults. They introduce consensus guidelines on environmental protective measures, microbial prophylaxis, post discharge precautions, which are especially important in the COVID-19 era, and vaccinations. Boelens et al. provide an overview of the association between specific conditioning agents and immune reconstitution (IR) post-HCT. Given the correlation between IR and outcome, they discuss a rationale for selection of a more standardized parameter set to monitor immune recovery that could be further studied and applied more widely. Additionally, Winger et al. address an important and often overlooked aspect of HCT which is the reconciliation of medications in the pre-HCT period to avoid harmful drug-drug interactions or overlapping toxicities with conditioning agents. As part of their report, they provide a very practical and extensive appendix with timelines for discontinuation or modification of common drugs prior to initiation of conditioning.

Relapse remains the primary cause of failure following HCT. Strategies to enhance graft-versus-leukemia (GVL) without increasing GvHD have been a major focus of research for decades. Summers et al. discuss the use of minor H antigens as T cell targets for augmenting GVL. Most minor H antigens are expressed ubiquitously, including on epithelial tissues, and as such can be recognized by donor T cells resulting in GvHD. This report focuses on donor-derived T cell responses against minor H antigens with hematopoietic-restricted expression. Following HCT, these hematopoietic-restricted minor H antigens present on residual recipient malignant hematopoietic cells can serve as tumor-specific antigens for donor T cells and thus be targets for selective GVL. This novel approach may hold promise for preventing relapse if given with the stem cell graft or as cell therapy for treating minimal residual disease (MRD) post-HCT. Wang et al. discuss the importance of monitoring MRD over the long treatment course of pediatric acute lymphoblastic leukemia (ALL). They followed over one thousand ALL patients documenting MRD re-emergence in ~15%. Three fourths of patients with MRD re-emergence continued to receive maintenance chemotherapy while 25% underwent mostly haplo-HCT. The HCT group had significantly better outcomes than the patients continuing with chemotherapy.

The final two papers in this series are disease specific. Krishnamurti discusses the complex topic of HCT for patients with sickle cell disease (SCD). He addresses the importance of careful patient selection considering the intricate compromise between the possibility of ameliorating SCD manifestations and early or late complications of HCT. Moreover, careful selection of donor, and choice of conditioning and GvHD prophylaxis regimens are examined as well as pre-HCT evaluation and post-HCT management of the unique complications encountered in patients with SCD. Ussowicz et al. performed a retrospective analysis of patients with high-risk neuroblastoma treated with MIBG I [131] therapy most of who then received high-dose chemotherapy with stem cell rescue. Their results suggest that MIBG I [131] may have utility pre-HCT and may warrant further study.

This collection of original and review articles addresses new developments in this exciting and very diverse field. Despite ongoing challenges, much hope exists in developing more effective and safer approaches to treat both malignant and none malignant disorders with cell therapies and transplantation.

## Author Contributions

All authors listed contributed to the editorial and approved the submitted version.

## Conflict of Interest

PH is a co-founder and on the board of directors of Mana Therapeutics, is on the scientific advisory board of Cellevolve, and on an advisory board of Maxcyte. The remaining authors declare that the research was conducted in the absence of any commercial or financial relationships that could be construed as a potential conflict of interest.

## Publisher's Note

All claims expressed in this article are solely those of the authors and do not necessarily represent those of their affiliated organizations, or those of the publisher, the editors and the reviewers. Any product that may be evaluated in this article, or claim that may be made by its manufacturer, is not guaranteed or endorsed by the publisher.

## References

[1] Wagner JE Ballen KK Zhang MJ Allbee-Johnson M Karanes C Milano F. Comparison of haploidentical and umbilical cord blood transplantation after myeloablative conditioning. Blood Adv. (2021) 5:4064–72. 10.1182/bloodadvances.2021004462 34461630 PMC8945645

[2] Bazarbachi A Labopin M Blaise D Forcade E Socie G Berceanu A. Comparable outcomes of haploidentical transplant with TBF conditioning versus matched unrelated donor with fludarabine/busulfan conditioning for acute myeloid leukemia. Bone Marrow Transplant. (2021) 56:622–34. 10.1038/s41409-020-01074-z 33020591

[3] Shah RM. Contemporary haploidentical stem cell transplant strategies in children with hematological malignancies. Bone Marrow Transplant. (2021) 56:1518–34. 10.1038/s41409-021-01246-5 33674791

[4] Bertaina A Zecca M Buldini B Sacchi N Algeri M Saglio F. Unrelated donor vs. HLA-haploidentical alpha/beta T-cell- and B-cell-depleted HSCT in children with acute leukemia. Blood. (2018) 132:2594–607. 10.1182/blood-2018-07-861575 30348653

[5] Katsanis E Sapp LN Varner N Koza S Stea B Zeng Y. Haploidentical bone marrow transplantation with post-transplant cyclophosphamide/bendamustine in pediatric and young adult patients with hematologic malignancies. Biol Blood Marrow Transplant. (2018) 24:2034–9. 10.1016/j.bbmt.2018.06.007 29908231 PMC7556327

